# Supplementary material for: Rapid Assessment of COVID-19 Mortality Risk with GASS Classifiers
Source: Biomedicines. 2023 Mar 9;11(3):831. doi: 10.3390/biomedicines11030831 (PMC10045158; doi:10.3390/biomedicines11030831)
Supplement: Supplementary file 1 [file biomedicines-11-00831-s001.zip › biomedicines-2142301-supplementary.pdf]

	0	+1	+2	+3	+4
**Age (years)**	<45	45-60	61-74	75-89	≥90
**Sex**	Female	Male			
**RR (bpm)**	<20	20-30	>30		
**PaO2/FiO2 ratio**	>300	200-300	100-199	<100	
**Lymphocytes count (n/mmc)**	>1000	500-999	<500		
**D-Dimer (mg/L FEU)**	<0.5	0.5-2.0	>2.0		
**CKI**	Moderate	Severe			
**eGFR (ml/min)**	>60	45-60	30-44	<30	
**Troponin (ng/ml)**	<20	20-50	>50		
**BNP (pg/ml)**	<100	>100			
**CRP (mg/dl)**	0-4.9	5.0-10.0	>10.0		

Supplementary Table S1 - GASS score items – RR= Respiratory Rate; bpm= breaths per minute; CKI= Chronic Kidney Injury; eGFR= estimated Glomerular Filtration Rate; BNP= Brain Natriuretic Peptide; CRP= C Reactive Protein.

	+1	+2	+3	+6
**Coronary Artery Disease**	*			
**Congestive Heart Failure**	*			
**Chronic Pulmonary Disease**	*			
**Peptic Ulcer Disease**	*			
**Peripheral Vascular Disease**	*			
**Mild Liver Disease**	*			
**Cerebrovascular Disease**	*			
**Connective Tissue Disease**	*			
**Diabetes**	*			
**Dementia**	*			
**Hemiplegia**		*		
**Moderate-to-Severe Renal Disease**		*		
**Diabetes with End-organ Damage**		*		
**Any Prior Tumour (within 5 yrs of Diagnosis)**		*		
**Leukaemia**		*		
**Lymphoma**		*		
**Moderate-to-Severe Liver Disease**			*	
**Metastatic Solid Tumour**				*
**AIDS (not only HIV Positive)**				*

Supplementary Table S2 - Charlson Comorbidity Index items – AIDS= Acquired ImmunoDeficiency Syndrome.

Variable	IoCp (N=76)	NIoCp (N=174)	*p*
Men	50 (65.8)	87 (50.0)	
Women	26 (34.2)	87 (50.0)	0.021
Age <45	3 (3.9)	25 (14.4)	0.016
Age 45-60	13 (17.1)	36 (20.7)	0.51
Age 61-74	29 (38.2)	33 (19.0)	0.001
Age 75-89	31 (40.8)	65 (37.4)	0.61
Age ≥90	0 (0.0)	15 (8.6)	0.008
RR <20 bpm	11 (14.5)	69 (39.7)	<0.001
RR 20-30 bpm	29 (38.2)	60 (34.5)	0.61
RR >30 bpm	19 (25.0)	4 (2.3)	<0.001
PaO2/fiO2 ratio >300	9 (11.8)	77 (44.3)	<0.001
PaO2/fiO2 ratio 200-300	18 (23.7)	40 (23.0)	0.81
PaO2/fiO2 ratio 100-199	23 (30.3)	8 (4.6)	<0.001
PaO2/fiO2 ratio <100	11(14.5)	3 (1.7)	<0.001
Lymphocytes >1000/mmc	32 (42.1)	91 (52.3)	0.11
Lymphocytes 500-999/mmc	34 (44.7)	70 (40.2)	0.58
Lymphocytes <500/mmc	10 (13.2)	11 (6.3)	0.08
CRP <5 mg/dl	31 (40.8)	88 (50.6)	0.16
CRP 5-10 mg/dl	12 (15.8)	47 (27.0)	0.06
CRP >10 mg/dl	32 (42.1)	37 (21.3)	0.001
eGFR >60 ml/min	54 (71.1)	118 (67.8)	0.55
eGFR 45-60 ml/min	12 (15.8)	19 (10.9)	0.27
eGFR 30-44 ml/min	5 (6.6)	13 (7.5)	0.81
eGFR <30 ml/min	4 (5.3)	23 (13.2)	0.06
D-dimer <0.5 mg/L FEU	8 (10.5)	48 (27.6)	0.002
D-dimer 0.5-2 mg/L FEU	47 (61.8)	78 (44.8)	0.035
D-dimer >2 mg/L FEU	21 (27.6)	39 (22.4)	0.51
HS TnI <20 ng/ml	42 (55.3)	107 (61.5)	0.49
HS TnI 20-50 ng/ml	13 (17.1)	29 (16.7)	0.85
HS TnI >50 ng/ml	14 (18.4)	27 (15.5)	0.50
BNP <100 pg/ml	41 (53.9)	95 (54.6)	0.94
BNP ≥100 pg/ml	27 (35.5)	64 (36.8)	0.94
Clinical GASS <5 pts	4 (5.3)	49 (28.2)	<0.001
Clinical GASS 5-10 pts	42 (55.3)	88 (50.6)	0.50
Clinical GASS >10 pts	30 (39.5)	37 (21.3)	0.003

Supplementary Table S3 - GASS score items – differences between groups in terms of Intensification of Care. IoCp = patients who needed Intensification of Care; NIoCp = patients who did not need Intensification of Care; RR = Respiratory Rate; CRP = C Reactive Protein; eGFR = estimated Glomerular Filtration Rate; HS TnI = High Sensitivity Troponin I; BNP = Brain Natriuretic Peptide; GASS = General Assessment of SARS-CoV-2 Severity.

Variable	Deceased (N=62)	Discharged (N=188)	*p*
Men	31 (50.0)	106 (56.4)	
Women	31 (50.0)	82 (43.6)	0.38
Age <45	0 (0.0)	28 (14.9)	0.001
Age 45-60	3 (4.8)	46 (24.5)	0.001
Age 61-74	9 (14.5)	53 (28.2)	0.031
Age 75-89	44 (71.0)	52 (27.7)	<0.001
Age ≥90	6 (9.7)	9 (4.8)	0.16
RR <20 bpm	14 (22.6)	66 (35.1)	0.06
RR 20-30 bpm	24 (38.7)	65 (34.6)	0.46
RR >30 bpm	9 (14.5)	14 (7.4)	0.08
PaO2/fiO2 ratio >300	10 (16.1)	76 (40.4)	0.001
PaO2/fiO2 ratio 200-300	17 (27.4)	41 (21.8)	0.12
PaO2/fiO2 ratio 100-199	11 (17.7)	20 (10.6)	0.052
PaO2/fiO2 ratio <100	4 (6.5)	10 (5.3)	0.55
Lymphocytes >1000/mmc	19 (30.6)	104 (55.3)	0.001
Lymphocytes 500-999/mmc	33 (53.2)	71 (37.8)	0.029
Lymphocytes <500/mmc	9 (14.5)	12 (6.4)	0.044
CRP <5 mg/dl	21 (33.9)	98 (52.1)	0.013
CRP 5-10 mg/dl	16 (25.8)	43 (22.9)	0.62
CRP >10 mg/dl	24 (38.7)	45 (23.9)	0.022
eGFR >60 ml/min	28 (45.2)	144 (76.6)	<0.001
eGFR 45-60 ml/min	11 (17.7)	20 (10.6)	0.13
eGFR 30-44 ml/min	7 (11.3)	11 (5.9)	0.14
eGFR <30 ml/min	15 (24.2)	12 (6.4)	<0.001
D-dimer <0.5 mg/L FEU	4 (6.5)	52 (27.7)	0.001
D-dimer 0.5-2 mg/L FEU	35 (56.5)	90 (47.9)	0.19
D-dimer >2 mg/L FEU	20 (32.3)	40 (21.3)	0.07
HS TnI <20 ng/ml	21 (33.9)	128 (68.1)	<0.001
HS TnI 20-50 ng/ml	16 (25.8)	26 (13.8)	0.030
HS TnI >50 ng/ml	21 (33.9)	20 (10.6)	<0.001
BNP <100 pg/ml	17 (27.4)	119 (63.3)	<0.001
BNP ≥100 pg/ml	42 (67.7)	49 (26.1)	<0.001
GASS <5 pts	2 (3.2)	51 (27.1)	0.53
GASS 5-10 pts	27 (43.5)	103 (54.8)	0.65
GASS >10 pts	33 (53.2)	34 (18.1)	0.82

Supplementary Table S4 - GASS score items – differences between groups in terms of in-hospital mortality. RR = Respiratory Rate; CRP = C Reactive Protein; eGFR = estimated Glomerular Filtration Rate; HS TnI = High Sensitivity Troponin I; BNP = Brain Natriuretic Peptide; GASS = General Assessment of SARS-CoV-2 Severity.

Variable	30-ddp (N=59)	30-dsp (N=191)	*p*
Men	28 (47.5)	109 (57.1)	
Women	31 (52.5)	82 (42.9)	0.20
Age <45	0 (0.0)	28 (14.7)	0.002
Age 45-60	3 (5.1)	46 (24.1)	0.001
Age 61-74	13 (22.0)	49 (25.7)	0.45
Age 75-89	37 (62.7)	59 (30.9)	<0.001
Age ≥90	6 (10.2)	9 (4.7)	0.12
RR <20 bpm	16 (27.1)	64 (33.5)	0.42
RR 20-30 bpm	20 (33.9)	69 (36.1)	0.89
RR >30 bpm	8 (13.6)	15 (7.9)	0.15
PaO2/fiO2 ratio >300	11 (18.6)	75 (39.3)	0.007
PaO2/fiO2 ratio 200-300	15 (25.4)	43 (22.5)	0.36
PaO2/fiO2 ratio 100-199	10 (16.9)	21 (11.0)	0.12
PaO2/fiO2 ratio <100	5 (8.5)	9 (4.7)	0.19
Lymphocytes >1000/mmc	21 (35.6)	102 (53.4)	0.018
Lymphocytes 500-999/mmc	29 (49.2)	75 (39.3)	0.16
Lymphocytes <500/mmc	8 (13.6)	13 (6.8)	0.10
CRP <5 mg/dl	21 (35.6)	98 (51.3)	0.037
CRP 5-10 mg/dl	15 (25.4)	44 (23.0)	0.69
CRP >10 mg/dl	22 (37.3)	47 (24.6)	0.052
eGFR >60 ml/min	27 (45.8)	145 (75.9)	<0.001
eGFR 45-60 ml/min	12 (20.3)	19 (9.9)	0.031
eGFR 30-44 ml/min	5 (8.5)	13 (6.8)	0.65
eGFR <30 ml/min	14 (23.7)	13 (6.8)	<0.001
D-dimer <0.5 mg/L FEU	4 (6.8)	52 (27.2)	0.001
D-dimer 0.5-2 mg/L FEU	38 (64.4)	87 (45.5)	0.010
D-dimer >2 mg/L FEU	15 (25.4)	45 (23.6)	0.78
HS TnI <20 ng/ml	18 (30.5)	131 (68.6)	<0.001
HS TnI 20-50 ng/ml	16 (27.1)	26 (13.6)	0.015
HS TnI >50 ng/ml	21 (35.6)	20 (10.5)	<0.001
BNP <100 pg/ml	17 (28.8)	119 (62.3)	<0.001
BNP ≥100 pg/ml	39 (66.1)	52 (27.2)	<0.001
GASS <5 pts	2 (3.4)	51 (26.7)	<0.001
GASS 5-10 pts	26 (44.1)	104 (54.5)	0.16
GASS >10 pts	31 (52.5)	36 (18.8)	<0.001

Supplementary Table S5 - GASS score items – differences between groups in terms of 30-day mortality. 30-ddp = patients deceased within 30 days; 30-dsp = patients survived after 30 days; RR = Respiratory Rate; CRP = C Reactive Protein; eGFR = estimated Glomerular Filtration Rate; HS TnI = High Sensitivity Troponin I; BNP = Brain Natriuretic Peptide; GASS = General Assessment of SARS-CoV-2 Severity.
